# Clinical Notes

**Published:** 1883-11

**Authors:** S. Swan

**Affiliations:** New York


### CLINICAL NOTES.

S. SWAN, M. D., NEW YORK.

A writer in the August number of THE HOMŒPATHIC PHYSICIAN gives ONE case of epistaxis. I will also give one. In 1875 the writer was called to see a child who was "bleeding to death," and was asked "to come quick." I found a boy about six years old bleeding profusely from both nostrils bright blood. The father, who was holding him and wiping the blood away, was, as well as the child, saturated with blood—both much alarmed. The moment I took in the situation I called for one-half a glass of water, and put in a few pellets of ——— of the ——— attenuation (I omit the name of the remedy as it is unproven, and the potency, as many object to them as not being high, but both the name and potency are at the service of any physician sufficiently interested in the case to inquire for them), and gave a spoonful to the patient; walked across the room to the window, and as I sat down I heard the father remark, "The flow seems less," and immediately after he said, "It has stopped." The mother then told me that he had often suffered from nose-bleeding since he was a year old, and requested, as they were going out of town, that I would give them some of the remedy in case he should have an attack. I did so. A year since the mother informed me that they had never had occasion to open the phial, as he never since had had the nose-bleed.

Finding by subsequent cases that the remedy cured epistaxis, whether from the right or left nostril, or both, or if the blood was bright or dark, fluent or clotted, I have given it in all cases, and never yet failed to effect a rapid cure, and other physicians have done the same.

As to epistaxis relieving congestions, I notice that congestion, or rush of blood to the heart, is omitted.

An eminent physician in a neighboring State had the case of a lady who had severe congestion of the heart when excited, so as to cause suffocation. In one of these attacks he gave her one dose of ——— the ——— potency (name of drug and potency omitted for above reasons). Relief came in a few moments, and with it a sharp pain going up from the heart toward the throat, and soon after the left nostril commenced bleeding and bled a cupful. Since then she has been excited and angry, but the congestion of blood in the heart has never returned.

A physician had a sudden attack of rush of blood to the head with fullness as if the head would burst, face flushed, carotids distended and pulsating strongly, with dull and stupid feeling. He had sense enough to take a dose of the same remedy as in the previous case, and in a few moments was relieved by a flow of blood from both nostrils, about a small teacupful.

The remedy in these two cases was not the same as that given for epistaxis.

It is well to know that we have drugs which have such control over the circulation.

I said the above drugs were unproven; they have never been published, but are in the hands of a few who are not afraid to use nosodes or morbific products, knowing that if they cure it is because they are homœopathic and act according to the law of the similars.

